# Robot-assisted minimally invasive transforaminal lumbar interbody fusion with contralateral facet decortication: a novel technique for posterolateral fusion

**DOI:** 10.3171/2025.4.FOCVID2520

**Published:** 2025-07-01

**Authors:** Mahmudur Rahman, Mohamed Jalloh, Aditya Vedantam

**Affiliations:** Department of Neurosurgery, Medical College of Wisconsin, Milwaukee, Wisconsin

**Keywords:** transforaminal lumbar interbody fusion, minimally invasive surgery, facet decortication

## Abstract

In traditional minimally invasive transforaminal lumbar interbody fusion (MIS-TLIF), the primary fusion mass comes from the interbody space. In this case report of a 58-year-old female undergoing a MIS-TLIF for grade 2 spondylolisthesis, the authors demonstrate a novel MIS posterolateral fusion technique using robot-guided facet decortication. This method enhances the fusion surface area in patients with osteopenia or spondylolisthesis and advances robotic spine fusion strategies.

The video can be found here: https://stream.cadmore.media/r10.3171/2025.4.FOCVID2520

## Transcript

The authors present robot-assisted minimally invasive transforaminal lumbar interbody fusion with contralateral facet decortication: a novel technique for posterolateral fusion.

### 0:31 Patient Presentation.

Fifty-eight-year-old female with 5-month history of low back pain radiating bilaterally to the posterolateral hips and feet with numbness and tingling. Pain worsened with ambulation and improved with sitting. She had intermittent urinary hesitancy but no bowel incontinence. Conservative measures were minimally effective, and an epidural provided only transient relief. Ambulation was significantly limited at the time of her presentation. Past medical history is significant for hypothyroidism, hyperlipidemia, and osteopenia. Past surgical history included a C3–6 ACDF for degenerative cervical myelopathy.

### 1:13 Physical Examination.

The physical exam showed an antalgic gait, and further gait analysis was limited by pain. Patient had full strength bilaterally in upper and lower extremities. However, she had hyperreflexia on her Achilles and patellar tendons and a positive FABER test bilaterally. She also had a positive straight leg test on her right. Lateral x-ray in neutral and standing position demonstrated a 1.5-cm grade 2 anterolisthesis at L4 and L5, along with a stable grade 1 retrolisthesis at L1–2. Flexion/extension views in Exhibit B reveal no significant motion instability. T2-weighted sagittal MRI on the left shows multilevel degenerative spondylosis that is most significant at L5–S1. At L4–5, we again see the grade 2 anterolisthesis, but now reveal severe canal stenosis, causing cauda equina compression and L5 nerve root impingement on the subarticular recesses. Additionally, moderate foraminal stenosis is seen at L4–5 and L5–S1, especially on the left side. In summary, we have an older female presenting with bilateral radiculopathy secondary to spondylolisthesis and L4–5 stenosis. Despite conservative measures, she continued to have debilitating leg and back pain and ultimately consented to an MIS-TLIF.

### 2:41 Operative Plan.

The operative plan for this patient included a minimally invasive right L4–5 TLIF with an expandable cage, posterolateral arthrodesis at L4–5 with allograft, bilateral L4 laminotomy and left medial facetectomy, and robot-assisted pedicle screw placement at L4 and L5. This preoperative thin-section CT without contrast was obtained to accurately register the patient’s anatomy with intraoperative fluoroscopy for accurate robotic navigation.

### 3:13 Positioning and Preparation.

After induction of general anesthesia, the patient was positioned prone on a Jackson table with all pressure points carefully padded and the field prepped and draped. An iliac pin was then placed in the left PSIS for robotic registration. AP and oblique fluoroscopy images were obtained to register the patient’s anatomy with her preoperative CT scan. The robot was then connected to the iliac pin, and accuracy of robotic navigation was verified.

### 3:43 Left-Sided Screw Placement and Facet Decortication.

Using the preplanned trajectories on the robotic navigation system, the left L4 and L5 pedicles were cannulated percutaneously, and screws were placed. The robot was used to guide a navigated cannula down to the left L4–5 facet joint. A specialized facet decortication tool (with a 1.5-mm safety margin) was used to carefully decorticate the joint without encroaching on the canal or foramen. Allograft was packed through the cannula into the decorticated joint space to establish a posterolateral fusion bed.

### 4:32 Right-Sided Access and Tubular Retractor Placement.

On the right side, L4 and L5 pedicle screw trajectories were mapped and cannulated under robotic navigations, and K-wires were placed in anticipation of later screw insertion. A 20-mm tubular retractor was docked on the right L4–5 facet and confirmed with navigation.

### 4:52 Decompression and TLIF.

Once the tubular retractor was docked on the facet, a thin layer of muscles were dissected to reveal the superior and inferior articular processes. A right-sided facetectomy and laminotomy was performed to decompress the exiting nerve root. Contralateral laminotomy was achieved by angling the tube across midline and removing the ligamentum flavum to decompress the thecal sac adequately. A standard anulotomy and discectomy was performed at L4 and L5, followed by a thorough disc space preparation with shavers and rongeurs. Autograft and allograft were packed into the interspace, and a 9-mm cage was inserted and expanded under fluoroscopy. Additional graft was postpacked around the cage to promote fusion.

### 5:44 Right-Sided Screw Placement.

Right L4 and L5 screws were then placed over the existing K-wires. Rods were subsequently inserted and the screws were used to gently reduce the L4–5 spondylolisthesis. Fluoroscopy images confirm the restoration of lumbar lordosis and correction of spondylolisthesis.

### 6:04 Postoperative Course.

The patient successfully recovered from anesthesia and was subsequently transferred to the floor. She reported improvement in her preoperative symptoms and was discharged on postoperative day 1. In her 1-week follow-up, she reported pain-free ambulation and was contemplating returning to virtual work. In postoperative week 6, she had minimal foot tingling and was back to full activity with lifting restrictions. Week 6 postoperative imaging showed stable hardware with no complications and an improvement in her spondylolisthesis when compared to her preoperative imaging.

### 6:41 Clinical Pearls.

The facet decortication tool allows contralateral facet decortication and graft placement using a minimally invasive approach. This enables a posterolateral arthrodesis to supplement the MIS-TLIF. By matching the trajectory angle of the inferior screw so that it is parallel to the pedicle screw, the same incision can be used for the decortication tool. If the facet joint is large, plan multiple trajectories to decorticate the entire joint surface. This enlarges the fusion surface area. In traditional MIS-TLIF, the primary fusion mass comes from the interbody space.^1–3^ By adding facet decortication on the contralateral side, you gain an additional posterolateral fusion bed.^4,5^ This is particularly valuable in patients with osteopenia or spondylolisthesis. This technique is versatile because it can be used whenever percutaneous screws are placed using the robot to ensure posterolateral arthrodesis. By integrating robotic navigation, we demonstrate the ability for robot-guided osseous drilling that could lead to further advances in semiautomated robotic surgery.^6^

## Disclosures

The authors report no conflict of interest concerning the materials or methods used in this study or the findings specified in this publication.

## Author Contributions

Primary surgeon: Vedantam. Editing and drafting the video and abstract: all authors. Critically revising the work: all authors. Reviewed submitted version of the work: all authors. Approved the final version of the work on behalf of all authors: Vedantam. Supervision: Vedantam, Jalloh.

## Supplemental Information

### Patient Informed Consent

The necessary patient informed consent was obtained in this study.
